# Whole-genome sequencing of the ant *Crematogaster osakensis* (Hymenoptera: Formicidae: Myrmicinae)

**DOI:** 10.1093/dnares/dsaf012

**Published:** 2025-05-20

**Authors:** Ayako Gotoh, Atsushi Toyoda, Takahiro Yamabe, Takehiko Itoh

**Affiliations:** Department of Biology, Faculty of Science and Engineering, Konan University, Kobe 658-8501, Japan; Institute for Integrative Neurobiology, Konan University, Kobe 658-8501, Japan; Suntory Rising Stars Encouragement Program in Life Sciences (SunRiSE), Kyoto 619-0284, Japan; Department of Genomics and Evolutionary Biology, and Advanced Genomics Center, National Institute of Genetics, Shizuoka 411-8540, Japan; School of Life Science and Technology, Institute of Science Tokyo, Tokyo, 152-8550, Japan; School of Life Science and Technology, Institute of Science Tokyo, Tokyo, 152-8550, Japan

**Keywords:** ants, *Crematogaster osakensis*, whole-genome sequencing

## Abstract

The ant *Crematogaster osakensis* (Hymenoptera: Formicidae: Myrmicinae) serves as a valuable model organism for investigating queen-specific traits, such as reproductive capacity and longevity, at the cellular and molecular levels. To support future research on queen traits using molecular techniques, including genome editing and multi-omics data analyses, we performed whole-genome sequencing of this species. The genome size of *C*. *osakensis* was estimated to be 284 Mb, with a heterozygosity of 0.26%. Annotation revealed the presence of 16,053 protein-coding genes. Furthermore, using the coding sequences annotated in this study, we reanalyzed previously obtained transcriptome data to identify highly expressed genes in the reproductive organs of queens and males. This data will contribute to a deeper understanding of the genetic mechanisms underlying reproductive strategies in ants.

## 1. Introduction

Ants are a significant ecological group owing to their substantial biomass.^1^ The evolution of highly advanced eusociality has significantly contributed to the ecological success of ants. In most ant species, there is a strict reproductive division of labor between queens and workers. Sterile workers perform colony tasks such as foraging and brood care. They are usually smaller than queens, lack wings, and have either absent or reduced reproductive organs.^2^ In contrast, queens, known for their larger body size, have wings (which are lost after mating) and well-developed reproductive organs. They primarily focus on egg production throughout their long lifespan, which can exceed 10 yr in many species. This longevity in ant queens challenges the evolutionary theory of aging seen in solitary species, where there is typically a trade-off between fecundity and longevity.^3^

Obtaining an adequate number of queens for experiments poses a significant challenge in most ant species. Consequently, investigating the cellular mechanisms associated with queen-specific traits, including morphogenesis, social behavior, reproductive ability, and longevity, becomes difficult. However, *Crematogaster osakensis* serves as an excellent model species for such studies due to its suitability for the collection of a large number of queens shortly after a nuptial flight (mating flight). Additionally, these queens establish colonies collectively and maintain polygyny even post-worker emergence (primary polygyny),^4^ enabling the housing of multiple queens in a single rearing case and thereby reducing the maintenance effort per queen. Our laboratory records indicate that the queen lifespan of this species surpasses 10 yr, with queens continuing to lay eggs. The longest-lived queens were collected in September 2012 after a mating flight and are still producing daughter workers from fertilized eggs as of June 2025. *Crematogaster osakensis* queens in our laboratory have been utilized for research focusing on the molecular and physiological mechanisms involved in long-term (equivalent to their long lifespan) sperm storage.^5–7^ This species also holds promise for investigating other queen-specific proximate trait mechanisms. To establish *C. osakensis* as a model organism for exploring the molecular and cellular mechanisms of queen-specific traits, whole-genome sequencing is crucial for subsequent analyses, such as genome editing and multi-omics approaches.

In this study, the genome of *C*. *osakensis* was sequenced and assembled using a combination of long-read and short-read technologies, followed by gene annotation. In a previous study, highly expressed genes in the queen and male reproductive organs, specifically the spermatheca and the male accessory gland, were identified by RNA-seq analyses using a reference sequence derived from *de novo* assembly.^5,8^ Subsequently, *in situ* hybridization was performed to determine the spatial expression patterns of these genes, leading to the identification of 12 genes specifically expressed in the spermatheca,^5^ among which 3 were also expressed in the male accessory gland.^8^ Here, we reanalyzed the previously obtained transcriptome data^5,8^ using the coding sequences annotated in this study as a reference to reidentify highly expressed genes in the queen spermatheca and the male accessory gland. Additionally, the information on spatial gene expression patterns previously reported^5^ was integrated with the genes annotated in this study.

## 2. Materials and methods

### 2.1 Sample collection

*Crematogaster osakensis* queens were collected after a mating flight in the Kagawa Prefecture in western Japan in September 2021. The queens were housed in plastic cases with moistened plaster bases at 25 °C for 22 days. Subsequently, the queens underwent 3 washes with sterilized water. Following this, their gut, poison gland, and spermatheca were removed, and each queen was then frozen at −80 °C.

### 2.2 Genome preparation

Genomic DNA was extracted from a single *C. osakensis* queen using a Genomic-tip Kit (QIAGEN, Hilden, Germany). The concentration and size of the genomic DNA extracted were assessed using a Qubit 4 Fluorometer (Thermo Fisher Scientific, MA, United States) and a Femto Pulse system (Agilent Technologies, CA, United States). A total of approximately 480 ng of genomic DNA was obtained with a concentration of 4.8 ng/µL.

### 2.3 Genome sequencing and assembly

Whole-genome shotgun sequencing was performed using the PacBio and Illumina sequencing platforms. For PacBio long-read sequencing, a continuous long-read (CLR) library was generated with a SMRTbell Express Template Prep Kit 2.0 (Pacific Biosciences, CA, United States) and sequenced on the PacBio Sequel II system using a Sequel II Binding Kit 2.0 and Sequencing Kit 2.0 (Pacific Biosciences, CA, United States) for 20 h. The raw data were processed with the PacBio SMRT Link v10.1.0.119549 program, yielding 40.6 Gb of CLR data from one 8M SMRT Cell. For Illumina sequencing, a paired-end library was prepared using an Illumina DNA PCR-Free Library Prep Tagmentation Kit (Illumina, CA, United States) and sequenced on the NovaSeq 6000 system (Illumina, CA, United States) with a read length of 2 × 150 bp, generating a total of 35.6 Gb of paired-end reads.

A 32-mer frequency analysis from the Illumina reads was performed using Jellyfish v2.3.0.^9^ Based on the results, estimates for genome size and heterozygosity were obtained through Genomescope 2.0.^10^ The PacBio CLR reads were assembled with NextDenovo v2.5.0.^11^ Since there was no significant discrepancy between the total length of the assembly results and the estimated genome size, no additional post-processing, such as purging, was performed. Polishing was performed using NextPolish^12^ with both PacBio CLR and Illumina reads. Simultaneously, the mitochondrial genome was assembled using GetOrganelle^13^ with Illumina reads. Finally, bacterial and mitochondrial sequences were removed from the genome assembly constructed from PacBio reads by conducting a BLASTN^14^ search against the NCBI nt database and the assembled mitochondrial genome. The assembly quality was assessed using BUSCO v5.4.2^15^ in genome mode against the endopterygota_odb10 dataset.

### 2.4 Gene annotation

Gene annotation was conducted by integrating 3 prediction methods using the GINGER^16^ pipeline: RNA-seq-based predictions, homology-based predictions against gene sequences of closely related species, and ab initio predictions. In the RNA-seq-based method, RNA-seq reads (Accession No. DRA005506)^5^ were mapped to the genome using HISAT v2.2.1,^17^ followed by mapping-based predictions using StringTie v2.2.0.^18^ Furthermore, transcriptome assembly was performed with Trinity v2.1.0^19^ and Oases v2.09^20^ using the same RNA-seq reads, and *de novo* assembly-based predictions were made by spliced-mapping the assembled transcripts to the genome using GMAP v2015-09-29.^21^ For the homology-based method, predictions were made by spliced-aligning protein-coding gene sequences from 12 ant species, including *Acromyrmex echinatior*, *Atta colombica*, *Cyphomyrmex costatus, Monomorium pharaonis, Pogonomyrmex barbatus, Solenopsis invicta, Temnothorax curvispinosus, Trachymyrmex cornetzi* (currently *Paratrachymyrmex cornetzi*)*, Trachymyrmex septentrionalis, Trachymyrmex zeteki* (currently *Mycetomoellerius zeteki*)*, Vollenhovia emeryi,* and *Wasmannia auropunctata*, using Spaln v2.3.3.^22^ Augustus v3.3^23^ was used for ab initio-based gene structure prediction. A set of 1,000 randomly selected RNA-seq-based predicted genes was used to train the gene model, which was then used to predict gene structure. The results predicted by the abovementioned 3 methods were integrated using the GINGER pipeline. The final integrated results were evaluated using BUSCO v5.4.2 in protein mode against the endopterygota_odb10 dataset.

The amino acid sequences translated from the predicted genes were subjected to a BLASTP homology search against the protein databases of *Drosophila melanogaster* (fruit fly), *Apis mellifera* (honeybee), and *T. curvispinosus* (ant) with an e-value cutoff of 1 × 10^−5^. The best-hit genes identified in each database are summarized in Table S1.

### 2.5 Reanalyzing previous transcriptome data

RNA-seq short reads generated previously from Illumine HiSeq2000^5,8^ were mapped to the coding sequences annotated in this study using Bowtie2 v2.5.2. Subsequently, the Salmon v1.10.0 alignment-based mode was utilized to obtain abundance estimates, and the Salmon output was summarized using tximport v1.30.0. Genes with very low expression levels, defined as fragments per kilobase of exon per million mapped reads (FPKM) values below 0.5 in all 23 RNA-sequencing samples,^5,8^ were excluded. Differential expression analyses were conducted on 14,110 genes across various comparisons: between the spermatheca and body samples at 1 wk and 1 yr after mating, between mated and unmated spermatheca in queens, and between accessory gland and body samples in males. Each tissue had 3 biological replicates, and the analyses were performed using DESeq2 v1.42.0.^24^ Significant expressed genes were identified based on a false discovery rate (FDR) < 0.01 and |log_2_ fold change| ≥ 1.

To integrate information on spatial gene expression patterns previously reported^5^ with annotated genes in this study, the sequences of 128 *in situ* hybridization probes from the previous study^5^ were compared with the coding sequences annotated in this study using BLASTN (e-value < 1 × 10^−100^). To detect potential matches outside annotated regions, the probe sequences were also compared with the whole-genome sequences using BLASTN (e-value < 1 × 10^−100^). In addition, the coding sequences from the previous RNA-seq study,^5^ which were used to design the *in situ* hybridization probes, and their translated protein sequences were compared with the annotated coding sequences and their translated protein sequences in this study using BLASTN and BLASTP (e-value < 1 ×10^−100^), respectively.

## 3. Results and discussion

### 3.1 Genome assembly and gene annotation

**Fig. 1.**
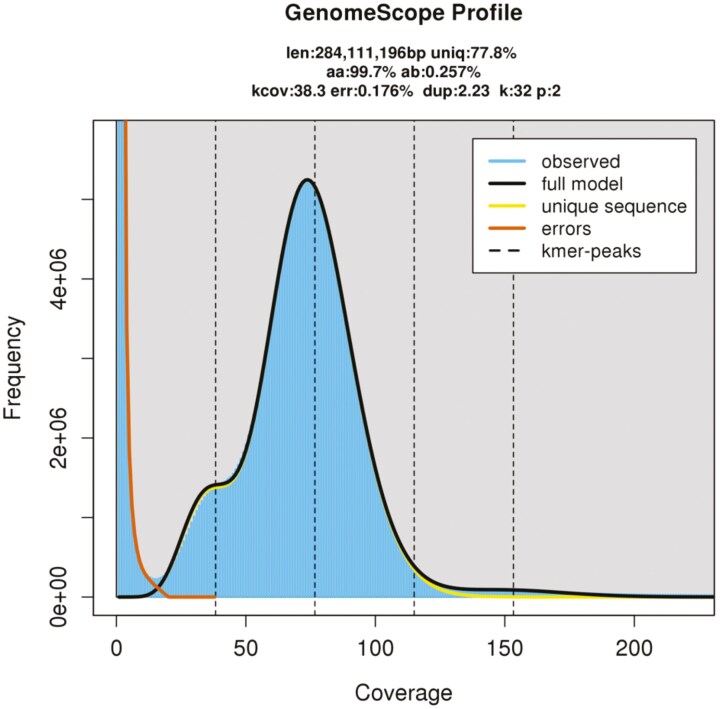
32-mer analysis of *Crematogaster osakensis* genome. The estimated haploid genome size was 284.1 Mb with 0.26% heterozygosity.

The Genomescope 2.0 analysis results are shown in Fig. 1. The estimated genome size and heterozygosity were 284 Mb and 0.26%, respectively. The nuclear genome assembly results are detailed in Table 1, revealing a highly contiguous genome comprising 154 scaffolds with a total length of 292.0 Mb and an N50 of 10.5 Mb. Genome completeness was evaluated using BUSCO, indicating very high values: 96.9% Complete (of which 96.4% single-copy), 0.3% Fragmented, and 2.8% Missing, confirming the construction of a highly comprehensive genome. Furthermore, a circular mitochondrial genome of 16,519 bp was assembled.

**Table 1.** Assembly statistics of *Crematogaster osakensis* genome.

Genome assembly statistics	
#Scaffolds	154
Total scaffold length (bp)	291,968,976
Longest scaffold (bp)	20,689,006
Scaffold N50 (bp)	10,545,608
Scaffold L50	12
Gaps (bp)	0
BUSCO evaluation (v5.4.2, genome mode, endopterygota_odb10)	
Complete BUSCOs (%)	99.1
Single-copy BUSCOs (%)	98.2
Duplicated BUSCOs (%)	0.9
Fragmented BUSCOs (%)	0.2
Missing BUSCOs (%)	0.7

As a result of gene prediction using the GINGER pipeline, 16,053 protein-coding genes were predicted. This number is almost equivalent to the 15,668 RefSeq genes documented for the closely related species, *T. curvispinosus*. Furthermore, the evaluation using BUSCO showed 99.1% Complete (of which 98.2% were single), 0.2% Fragmented, and 0.7% Missing. These values not only surpassed the BUSCO scores obtained from the genome assembly but also marked a value nearly equal to the 99.3% Complete evaluation for *T. curvispinosus*, suggesting that highly accurate gene predictions were achieved.

### 3.2 Reanalysis of highly expressed genes in queen and male reproductive organs based on the genome information

As a result of reanalysis of previous transcriptome data,^5,8^ 1,463 and 990 genes were upregulated, while 2,498 and 2,306 genes were downregulated in the spermatheca samples compared with those in the body samples after 1 wk and 1 yr of mating, respectively (FDR < 0.01 and |log_2_ fold change| ≥ 1; Table S1). Additionally, 192 and 46 genes were up- and downregulated, respectively, in the spermatheca of inseminated queens compared with virgin queens. Similarly, 140 and 285 genes were increased and decreased, respectively, in the spermatheca at 1 yr compared with 1 wk after mating (FDR < 0.01 and |log_2_ fold change| ≥ 1; Table S1).

To integrate the previously reported *in situ* hybridization data with annotated genes obtained in this study, BLASTN searches were conducted using the 128 probe sequences derived from *de novo* assembled contigs against the annotated coding sequences of *C. osakensis*. Of these, 108 probes showed sequence homology to the annotated coding sequences (Table S2). Two gene IDs—C.osakensis_01585 and C.osakensis_09374—each corresponded to 2 different probe sequences. For example, both comp63322_c0_seq1.m.59242, which was specifically expressed in the spermathecal gland, and comp64346_c0_seq1.m.68928, which was expressed in the spermathecal gland, ovary, and midgut, were homologous to C.osakensis_01585 (Table 2). Among the 12 genes previously identified as spermatheca-specific expression by *in situ* hybridization,^5^ 10 were found to have homologs among the annotated genes, including the aforementioned C.osakensis_01585, in this study (Table 2), and all 10 were upregulated in the spermatheca relative to the body samples. Of the remaining 20 among 128 probe sequences, 19 were homologous to the genome but not to the annotated coding sequences. In 4 of these cases, the original contigs used for probe design also matched annotated coding sequences, suggesting that the probes may have been designed slightly outside the annotated coding regions. For the other 15 probe sequences, their original contigs used for probe design were not homologous to the coding sequences. Among these, 12 original contig sequences (used to design the probes)—including 2 spermatheca-specific expressed genes (comp71230_c1_seq1.m.377439 and comp71576_c7_seq1.m.430348)—were subjected to BLASTP searches against NCBI’s non-redundant (nr) protein database in the previous study, but no significant hits were obtained (Table S2). This result suggests that although these gene sequences were indeed present in the current genome assembly, their absence from existing databases likely led to their omission during the annotation process in this study, even though RNA-seq-based predictions were performed. One remaining probe sequence among 128 probe sequences did not show detectable similarity to either the genome or the annotated coding sequences at the nucleotide level. However, the corresponding contig exhibited sequence similarity to a predicted amino acid sequence in this study. This discrepancy may be due to minor sequence errors or assembly differences in the previous transcriptome data that prevented nucleotide-level alignment.

**Table 2.** List of spermatheca-specific expressed genes after reanalysis of the previous transcriptome data.

Gene ID	Homology to *Temnothorax curvispinosus* genes	Log_2_ fold change (spermatheca/body)		Expression pattern in queens (Gotoh et al.^5^)	Log_2_ fold change (accessory glands/body)^a^
		1 wk after mating	1 yr after mating		
C.osakensis_02883	Uncharacterized protein LOC112459141	9.7	9.7	Hilar columnar epithelial cells of spermatheca reservoir	NS
C.osakensis_01585	Mucin-5AC-like	8.7	10.0	Spermathecal gland (+ midgut and ovary)^b^	NS
C.osakensis_00390	Nicotinamidase-like	8.7	7.2	Spermathecal gland (central duct)	−3.9
C.osakensis_02323	Peroxidasin-like	7.0	6.9	Spermathecal gland	2.0
C.osakensis_07475	Ammonium transporter Rh type B-B isoform X1	4.4	4.8	Spermathecal gland	−7.1
C.osakensis_00911	Uncharacterized protein LOC112463818	4.2	3.2	Spermathecal gland	2.7
C.osakensis_06063	Multidrug resistance-associated protein 4-like	3.4	3.4	Spermathecal gland	−3.5
C.osakensis_00591	Proton-coupled amino acid transporter 1 isoform X2	3.3	3.6	Spermathecal gland	NS
C.osakensis_00886	Protein unc-13 homolog 4B isoform X6	2.7	2.6	Spermathecal gland	−2.3
C.osakensis_12094	Xanthine dehydrogenase-like	2.1	2.2	Spermathecal gland	−2.7

^a^NS shows that the expression is not significantly different between samples (FDR ≥ 0.01 and |log_2_ fold change| < 1).

^b^C.osakensis_01585 is homologous to 2 *i*n situ** hybridization probe sequences: 1 detected only in the spermathecal gland, and the other detected in the spermathecal gland, midgut, and ovary.

Furthermore, 1,426 genes were upregulated, and 2,954 genes were downregulated in the accessory glands compared to body samples from males. In a previous transcriptome analysis, 3 spermathecal gland-specific expressed genes in queens were found to be enriched in the male accessory gland.^8^ Two of these 3 genes, other than comp71230_c1_seq1.m.377439, were homologous to the C.osakensis_00911 and C.osakensis_02323 genes identified in this analysis and were enriched in the accessory gland relative to the body (Table 2).

When comparing previous and current analyses, the characteristics of the highly expressed genes remained consistent, despite differences in the analysis software used and variations in software versions. Consequently, the top candidate genes related to the long-term sperm storage mechanism in queens remained largely unchanged. For example, C.osakensis_01585, C.osakensis_00886, and C.osakensis_02883 appear to be crucial as they were identified as spermatheca-specific expressed genes^5^ and were also enriched in the proteome analysis of the spermathecal fluid of *Lasius japonicus* queen.^25^ Obtaining full-length gene sequences is experimentally laborious; however, whole-genome sequencing in this study significantly reduced this effort. In the future, utilizing *C. osakensis* queens as a research model has substantial potential for elucidating queen-specific phenomena, which could lead to substantial advancements in our understanding.

## Supplementary Material

dsaf012_suppl_Supplementary_Tables_S1-S2

## Data Availability

The accession number for the PacBio CLR sequencing data is DRR611971, and those for Illumina sequencing data are DRR611969 and DRR611970. The accession numbers for the *C. osakensis* assemblies are BAAFZW010000001-BAAFZW010000155.
